# The Rho-kinase inhibitor HA-1077 suppresses proliferation/migration and induces apoptosis of urothelial cancer cells

**DOI:** 10.1186/1471-2407-14-412

**Published:** 2014-06-07

**Authors:** Hideyuki Abe, Takao Kamai, Keitaro Hayashi, Naohiko Anzai, Hiromichi Shirataki, Tomoya Mizuno, Yoshiyuki Yamaguchi, Akinori Masuda, Hideo Yuki, Hironori Betsunoh, Masahiro Yashi, Yoshitatsu Fukabori, Ken-Ichiro Yoshida

**Affiliations:** 1 Department of Urology, Dokkyo Medical University, 880 Kitakobayashi, Mibu, Tochigi 321-0293, Japan; 2 Department of Pharmacology and Toxicology, Dokkyo Medical University, Mibu, Tochigi, Japan; 3 Department of Molecular and Cell Biology, Dokkyo Medical University, Mibu, Tochigi, Japan

## Abstract

**Background:**

Activation of Rho, one of the small GTPases, and its major downstream target Rho-kinase (ROCK) promotes the development and metastasis of cancer. We previously showed that elevation of Rho and ROCK expression was associated with tumor invasion, metastasis, and an unfavorable prognosis in patients with urothelial cancer of the bladder or upper urinary tract.

**Methods:**

We investigated the effects of a ROCK inhibitor on the growth, migration, and apoptosis of bladder cancer cells. We also examined phosphorylation of RhoA (RhoA activity) by measuring its GTP-bound active form and assessed the expression of ROCK to explore the underlying molecular mechanisms.

**Results:**

Lysophosphatidic acid (LPA) and geranylgeraniol (GGOH) induced an increase of cell proliferation and migration in association with promotion of RhoA activity and upregulation of ROCK expression. The ROCK inhibitor fasudil (HA-1077) suppressed cell proliferation and migration, and also induced apoptosis in a dose-dependent manner. HA-1077 dramatically suppressed the expression of ROCK-I and ROCK-II, but did not affect RhoA activity.

**Conclusions:**

These findings suggest that ROCK could be a potential molecular target for the treatment of urothelial cancer.

## Background

The standard treatment for muscle-invasive bladder cancer (MIBC) is radical cystectomy and bilateral pelvic lymph node dissection (PLND), while that for upper urinary tract cancer is radical nephroureterectomy and retroperitoneal lymph node dissection (RPLND). These radical procedures have become standard treatment over the past 30 years, but patients still have a relatively poor prognosis and the 5-year survival rate after surgery is less than 50% [1-3]. Although systemic chemotherapy with methotrexate, vinblastine, doxorubicin, and cisplatin (M-VAC) can reduce the tumor burden in patients with urothelial cancer, its influence on the prognosis is not very impressive [4]. Gemcitabine plus cisplatin (GC) has a better safety profile than M-VAC and may be considered as the first-line treatment for metastatic bladder cancer [5]. Some patients develop systemic metastases within a few years of curative resection. The most frequent sites of metastasis are the regional lymph nodes, liver, lungs, and bone [6], and the outlook for these patients is poor. Presumably, recurrence is due to occult micrometastasis at the time of surgery occurring via the rich lymphatic drainage of the bladder and upper urinary tract. Metastasis, i.e., tumor cell spread from the primary lesion to a distant site [7], is the major cause of cancer death. Various studies have shown that poorly differentiated cancer, muscle invasion, lymph node metastasis, and lymphovascular invasion are associated with recurrence of bladder cancer and are unfavorable prognostic factors. Therefore, it seems important to investigate the process of tumor cell dissemination.

Tumor cell migration is essential for metastasis, and migration involves rearrangement of the actin cytoskeleton. Accordingly, investigation of the regulation of actin cytoskeletal proteins could be important for understanding tumor metastasis. Members of the Rho family of small GTPases are involved in regulating a variety of cellular processes, including organization of the microfilament network, intercellular contact, and malignant transformation [8]. These cellular events are all interrelated. Specifically, certain subfamilies of Rho proteins are involved in regulating the actin cytoskeleton during the formation of stress fibers and focal adhesions within cells. The Rac subfamily regulates the formation of lamellipodia and membrane ruffles, while the Cdc42 subfamily regulates filopodia. Both lamellipodia and filopodia are seen at the advancing edge of motile cells, while retraction occurs on the opposite side [9,10], and these processes are accompanied by reorganization of the actin cytoskeleton. Rho-associated serine-threonine protein kinase (ROCK) [11,12] is one of the best characterized downstream effectors of Rho. ROCK is activated when it selectively binds to the active GTP-bound form of Rho, after which activated ROCK interacts with the actin cytoskeleton to promote stress fiber formation and the assembly of focal contacts [13,14].

GTPases from the Rho family have been linked to progression of human cancer, and the Rho/ROCK pathway is considered to be involved in tumor progression by regulating the actin cytoskeleton [15-17]. In fact, (R)-(+)-trans-N-(4-pyridyl)-4-(1-aminoethyl)-cyclohexanecarboxamide dihydrochloride (Y-27632) [18] is a specific ROCK inhibitor that suppresses tumor growth and metastasis, indicating that the Rho/ROCK pathway may be a good target for preventing tumor invasion and metastasis [19,20]. Thus, this pathway is an attractive molecular target for anticancer therapy. We previously reported that overexpression of Rho and ROCK proteins by bladder cancer and upper urinary tract cancer was associated with poorly differentiated histology, muscle invasion, lymph node metastasis, and shorter survival, indicating that the Rho/ROCK pathway is involved in the progression of urothelial cancer [21-23]. Accordingly, suppression of the Rho/ROCK pathway might potentially improve the outcome of patients with urothelial cancer.

Fasudil (HA-1077) was developed as a pharmacological ROCK inhibitor [24,25]. HA-1077 and its major active metabolite after oral administration (hydroxyfasudil) potently inhibit ROCK by promoting myosin light chain phosphorylation in vascular smooth muscle cells [26,27]. It has been reported that HA-1077 is effective for the treatment of cardiovascular disease, including coronary and cerebral vasospasm, arteriosclerosis/stenosis, ischemia/reperfusion injury, systemic hypertension, pulmonary hypertension, stroke, and heart failure [25]. Among the various ROCK inhibitors, HA1077 is the only clinically available one without obvious adverse effects [28]. HA-1077 has been recognized as a promising agent for preventing recurrent vasospasm of cerebral arteries after aneurysmal subarachnoid hemorrhage and its use is covered by the Japanese national health insurance system.

Because it inhibits the Rho/ROCK pathway, we investigated whether HA-1077 could block the proliferation and migration of bladder cancer cell lines or induce apoptosis of these cells. Our objective was to assess the value of the Rho/ROCK pathway as a molecular target for anticancer therapy. In this report, we discuss the clinical potential of HA-1077 for use in targeted cancer therapy.

## Methods

### Cell culture

Two human bladder cancer cell lines (5637 and UM-UC-3) were obtained from the American Type Culture Collection (ATCC, Rockville, MD). Cells were grown in RPMI-1640 medium supplemented with fetal bovine serum, 2 mM L-glutamine, 100 mg/mL streptomycin, and 100 units/mL penicillin (all from Life Technologies, Carlsbad, CA). Culture was performed at 37°C in a humidified atmosphere with 5% CO_2_. Adherent cells were detached from the culture dishes with trypsin/EDTA (Sigma, Deisenhofen, Germany).

### Inhibition of cell growth

5637 cells and UM-UC-3 cells (5 × 10^4^/well) were seeded into 96-well plates in serum-containing medium and were allowed to attach for 24 h. Then the medium was removed and replaced with new medium containing various concentrations of HA-1077. After being cultured for 72 h, the cells were incubated with 50 μL of 3-[4,5-dimethylthiazol-2-yl]-2,5-diphenyl tetrazolium bromide (MTT, 5 mg/mL; Sigma, St Louis, USA) for 1 h at 37.8°C. The formazan product was dissolved in 100 mL of DMSO and its absorbance was measured at a wavelength of 630 nm in a microplate reader. Proliferation of 5637 cells and UM-UC-3 cells was also assessed by quantitative ELISA based on the incorporation of BrdU during DNA synthesis (Roche Diagnostics, Mannheim, Germany), which was performed as reported previously [29]. The in vitro antiproliferative effect of HA-1077 was evaluated after incubation of cells in the presence of HA-1077 with or without lysophosphatidic acid (LPA) and geranylgeraniol (GGOH), which is an intermediate of the mevalonate pathway. Results were expressed as the ratio of the number of viable cells after incubation with HA-1077 to the number of cells in control cultures with PBS (a value of 100% was assigned to control cultures after incubation for 72 h). HA-1077 was kindly donated by Asahi Kasei Pharma (Tokyo, Japan). Each experiment was repeated five times.

### Clonogenic assay

Cells (100 cells/well) were seeded in 6-well plate. After cells adhered, HA-1077 (30 μM) or same volume of DMSO were added into medium. The medium and chemicals were freshly changed every two days. The cells were cultured for 8 days and stained with 6% glutaraldehyde and 0.5% crystal violet, as described previously [30,31]. Each experiment was repeated three times.

### Induction of apoptosis

Induction of apoptosis in bladder cancer cell lines by HA-1077 with or without LPA and GGOH was evaluated after incubation of 1 × 10^5^ cells for 24 hours in serum-free medium, followed by exchange of the medium for 10% FBS containing HA-1077 at its IC_50_ concentration. Cells were harvested by centrifugation and incubated for 24 hours at 4°C in 10 × fetal bovine serum. Then quantification of DNA fragmentation was done with an Apoptosis in situ Detection Kit (Wako Pure Chemical Industries, Osaka, Japan), and the average percentage was calculated for the three areas with the highest number of apoptotic cells among 500 cells in a single field at × 200 magnification. Apoptotic cells were identified by the terminal deoxynucleotidyl transferase-mediated deoxyuridine triphosphate biotin nick end-labeling (TUNEL) method using an in situ apoptosis detection Guava TUNEL kit (Merck Millipore, Darmstadt, Germany) [32]. Each experiment was repeated five times.

### Migration assay

The migration assay was performed using a modified Boyden chamber with a 24-well dish. Filters with 8-μm pores (Nucleopore Corp., Pleasanton, CA) were coated with Matrigel (40 mg; Collaborative Biomedical, Becton Dickinson Labware, San Jose, CA). Cells (2.5 × 10^4^) and were placed into 100 ml of complete RPMI medium in the upper chamber, while the lower chamber was filled with 1 mL of RPM1 medium containing LPA, GGOH, HA-1077, or BSA. After incubation for 48 hours, cells were fixed in methanol for 15 min and then stained with 0.05% crystal violet in PBS for 15 min. Cells on the upper side of each filter were removed with cotton swabs, and the filters were washed in PBS. Then the cells on the underside of each filter were counted under a microscope (type 090–135.001, Leica Microsystems, Wetzlar, Germany). The ratio of migrated cells to viable cells at control cells were set as 1.0 and the ratio of migrated cells to viable cells at treated cells were calculated as a percentage of the control. Clones were plated in triplicate for each experiment, and each experiment was repeated five times.

### RhoA activation assay and Western blotting

To measure the phosphorylated active RhoA (RhoA activity; i.e., its GTP-bound active form), we performed a Rho-binding domain (RBD) affinity precipitation assay for RhoA-GTP with a specific antibody targeting RhoA according to the manufacturer’s protocol (Cytoskeleton, BK036, Denver, CO) [23,33]. Active RhoA was precipitated from cell lysates (200 mg) with 15 mg of GST-RED (containing amino acids −8-89 of Rhotekin), which was expressed in Escherichia coli and bound to agarose beads. The precipitates were washed with washing buffer (50 mmol/L Tris [pH 7.2], 150 mmol/L NaCl, 10 mmol/L MgCl_2_, 0.1 mmol/L phenylmethylsulfonyl fluoride, 10 mg/mL aprotinin, and 10 mg/mL leupeptin). After adding the loading buffer and boiling for 5 min, the bound proteins were resolved on 12% polyacrylamide gel, transferred to a nitrocellulose membrane, and immunoblotted with the anti-RhoA antibody. After washing the pellet three times, the bound proteins were analyzed by Western blotting, as described previously. Briefly, proteins from total cell lysate (75 mg) or proteins obtained by trichloroacetic acid precipitation of conditioned medium harvested after 48 h of incubation (when the cells reached confluence) were separated by SDS-PAGE, followed by electrotransfer to a polyvinylidene difluoride membrane (Immobilon-P membrane; Millipore, Bedford, MA). After the membrane was blocked in a solution of 5% skim milk, 0.1% Tween 20, and PBS, the bound proteins were probed with primary antibodies directed against RhoA and β-actin (Santa Cruz Biotechnology, Santa Cruz, CA). Hela cells were used as the positive control as described previously [23]. Then the membranes were washed and incubated with horseradish peroxidase-conjugated secondary antibodies for 60 min. Antibody-bound protein bands were detected with enhanced chemiluminescence reagents (Amersham Pharmacia Biotech, Piscataway, NJ) and photographed with Kodak X-Omat Blue autoradiography film (Perkin Elmer Life Science, Boston, MA). In contrast to RhoA, we could not obtain commercial antibodies for phosphorylated active ROCK. Instead, we examined ROCK-I and ROCK-II protein expression using specific antibodies (sc-6055 for ROCK-I and sc-1851 for ROCK-II, each diluted 1:2000; Santa Cruz Biotechnology, Santa Cruz, CA). Bands of antibody-bound proteins were visualized by chemiluminescence, densitometry of the blotted membrane was done with a PDI imaging scanner (Agfa Japan, Tokyo), and the data were analyzed with NIH Image software. Expression of phosphorylated RhoA, ROCK-I, and ROCK-II was calculated relative to that of β-actin (set at 1.0) by densitometric analysis, as described previously [21,23,34,35]. Each experiment was repeated five times.

### Immunohistochemistry

Immunohistochemistry was done using the same specific antibodies as those for Western blotting, in order to support the data obtained by Western blotting as described previously [21,23]. The protein levels of RhoA activity, ROCK-I, and ROCK-II in the culture supernatants from the HA1077-treated bladder cancer cell lines and the control (non-treated bladder cancer cell lines) were measured. For each sample, 4 randomly selected areas were observed under high magnification and 100 tumor cells in each area were counted to calculate the proportion of positive cells.

### Statistical analysis

Data were analyzed by the Mann–Whitney *U* test for comparisons between two groups [21,23,35]. Because Boneferroni’s correction is generally employed for multiple comparisons, the Mann–Whitney *U* test was corrected by this method. In all analyses, a *P* value of less than 0.05 was considered significant. Data were analyzed with commercially available software.

## Results

**Figure 1:**
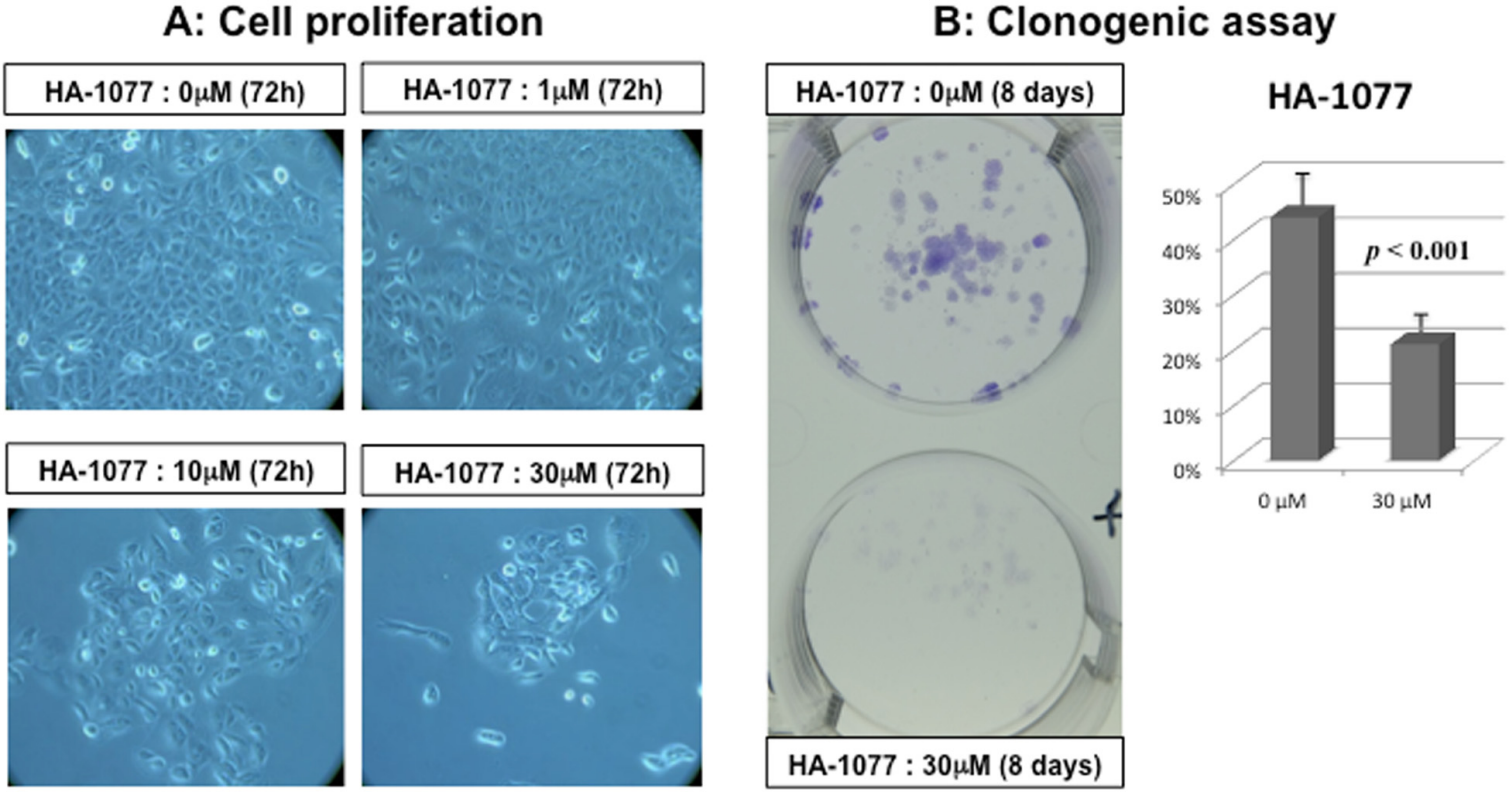
**The inhibitory effects of HA-1077 on cell proliferation.** Cell proliferation was inhibited by HA-1077 in 5637 cell **(A)**. In clonogenic assay **(B)**, HA-1077 significantly decreased the number of colonies compared with control cultures in 5637 cells.

Cell proliferation was inhibited by HA-1077 in a dose-dependent manner (Figure 1).In clonogenic assay, HA-1077 made significantly less number of colonies compared to control cultures (Figure 1B).

**Figure 2:**
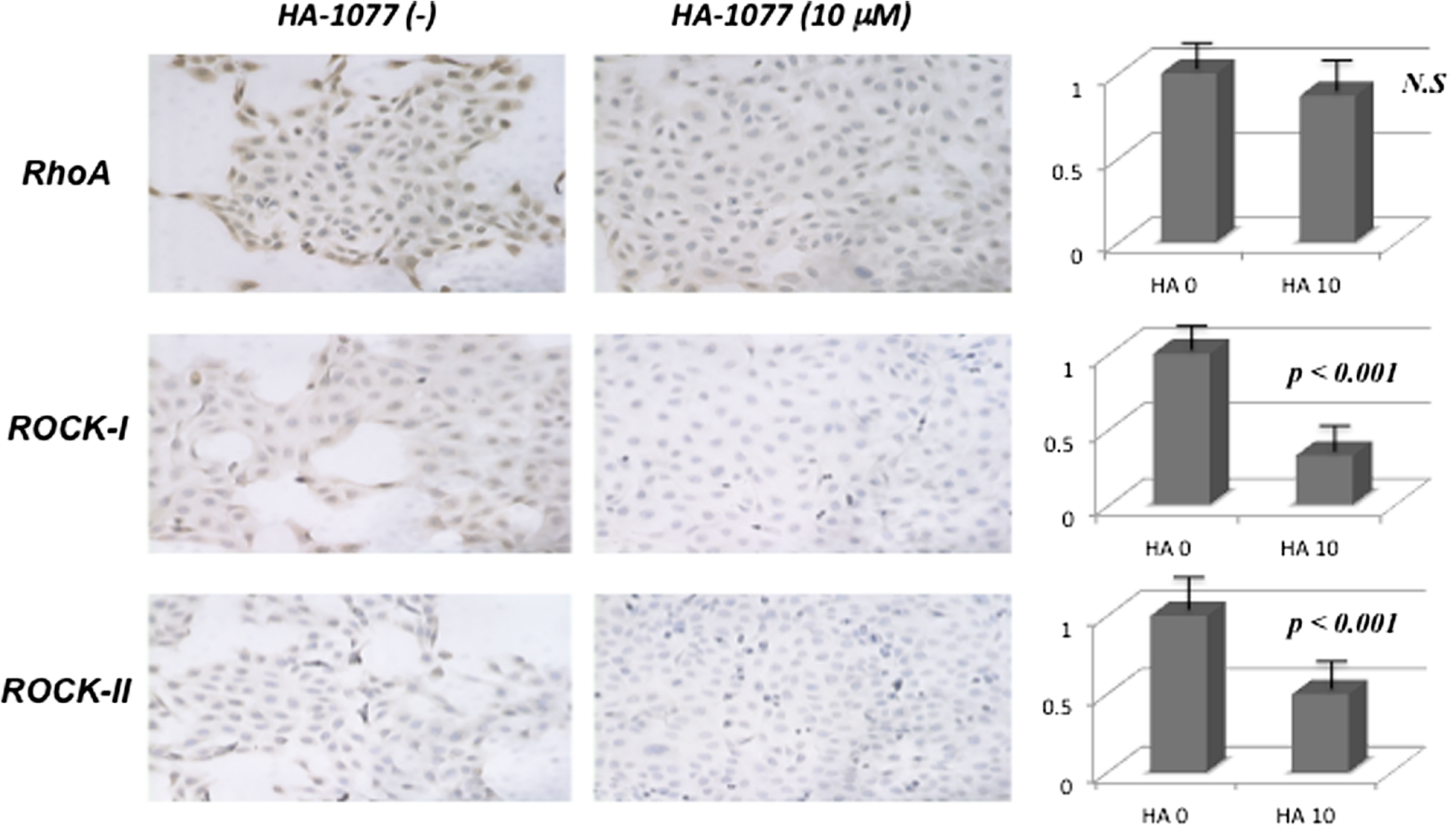
**Immunohistochemical staining using anti-RhoA, anti-ROCK-I and anti-ROCK-II monoclonal antibodies in 5637 (x20 magnification).** 70-80% of tumor cells showed moderate to strong cytoplasmic staining reaction for anti-RhoA antibody, and weak to moderate cytoplasmic staining for anti-ROCK-I and anti-ROCK-II antibodies. HA-1077 reduced the staining reactivity for anti-ROCK-I and anti-ROCK-II antibodies to very weak staining in only 20-30% of tumor cells, while it did not decrease the staining for anti-RhoA antibody with weak to moderate staining.

**Figure 3:**
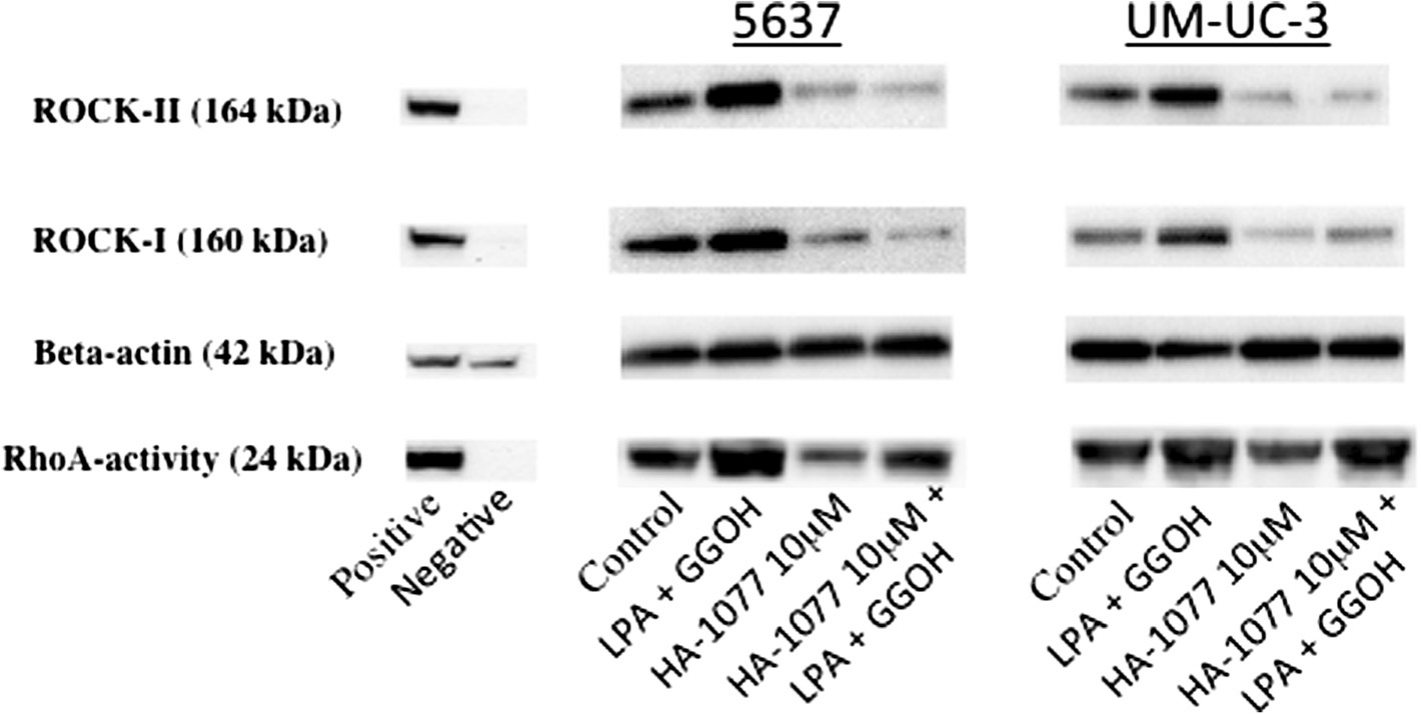
**Representative bands identified by Western blotting in 5637 and UM-UC-3 cells.** Expression of RhoA (24 kDa), ROCK-I (160 kDa), ROCK-II (164 kDa), and beta-actin (42 kDa). Hela cells were used as the positive control. In both cancer cells, LPA plus GGOH induced upregulation of RhoA activity, ROCK-I and ROCK-II. While HA-1077 significantly decreased ROCK-I and ROCK-II expression, this inhibitory effect of HA-1077 was not blocked by addition of LPA plus GGOH. HA-1077 did not influence RhoA activity.

On immunohistochemical analysis, the cytosolic compartment showed brown staining in most of the cancer cells, indicating high RhoA activity and high ROCK-I and ROCK-II protein levels, while the nuclei showed very weak staining (Figure 2). This staining pattern was identical to that detected in our previous study [21-23]. 70-80% of the cells from both bladder cancer cell lines showed moderate to strong cytoplasmic staining by anti-RhoA antibody, and weak to moderate cytoplasmic staining by anti-ROCK-I and anti-ROCK-II antibodies. HA-1077 reduced the level of reactivity with anti-ROCK-I and anti-ROCK-II antibodies to very weak staining in 20-30% of tumor cells, while the extent of staining for anti-RhoA antibody was not changed much, but its intensity was reduced to weak or moderate.Western blotting revealed that LPA and GGOH increased RhoA activity, as well as the expression of ROCK-I and ROCK-II (Figure 3). HA-1077 dramatically decreased the expression of ROCK-I and ROCK-II, and this decrease was not reversed by addition of LPA and GGOH. In contrast, HA-1077 did not reduce RhoA activity, while LPA and GGOH increased RhoA activity despite the addition of HA-1077.

### Inhibition of cell proliferation by HA-1077

**Figure 4:**
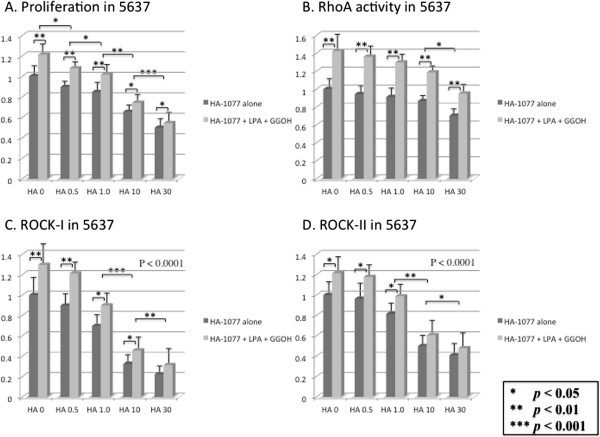
**Inhibiting effect of HA-1077 on proliferation and RhoA activity and ROCK expression in 5637 cell using the 3-(4,5-dimethyl-2-thiazolyl)-2,5diphenyl-2H-tetrazolium (MTT) assay.** The ratio of the cells treated with various doses (0.5–30 μM) of HA-1077 to control cells (HA-1077 0 μM) set as 1.0 were calculated. HA-1077 inhibited the growth of these cells in a dose-dependent manner **(A)**. HA-1077 did not reduce the RhoA activity **(B)**, but suppressed ROCK-I **(C)** and ROCK-II **(D)** in dose-dependent manner. The data show the 95% confidential interval.

**Figure 5:**
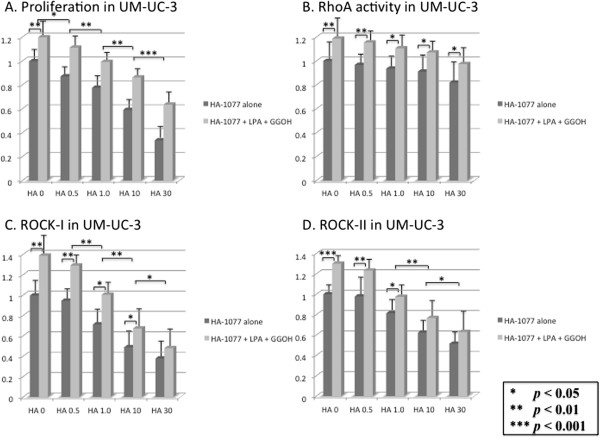
**Inhibiting effect of HA-1077 on proliferation and RhoA activity and ROCK expression in UM-UC-3 cell using the 3-(4,5-dimethyl-2-thiazolyl)-2,5diphenyl-2H-tetrazolium (MTT) assay.** The ratio of the cells treated with various doses (0.5–30 μM) of HA-1077 to control cells (HA-1077 0 μM) set as 1.0 were calculated. HA-1077 inhibited the growth of UM-UC-3 cell in a dose-dependent manner **(A)**. HA-1077 did not decrease the RhoA activity **(B)**, but inhibited ROCK-I **(C)** and ROCK-II **(D)** in dose-dependent manner. The data show the 95% confidential interval.

We examined the inhibitory effect of HA-1077 on the in vitro growth of human bladder cancer cell lines. Addition of LPA and GGOH increased cell proliferation along with upregulation of RhoA activity and elevation of expression of ROCK-I and ROCK-II (Figures 4 and 5). Cell proliferation was inhibited by HA-1077 in a dose-dependent manner, both when HA-1077 was added alone and when it was added in combination with LPA and GGOH (Figures 4A and 5A). Expression of ROCK-I and ROCK-II was significantly decreased by HA-1077 in a dose-dependent manner, but RhoA activity was only reduced slightly (Figures 4B-D, and 5B-D).On the other hand, comparison between cells treated by HA-1077 alone and those treated by HA-1077 in combination with LPA and GGOH revealed that RhoA activity was higher in the latter cells at each HA-1077 concentration (Figures 4B and 5B), while the difference in the expression of ROCK-I and ROCK-II gradually became smaller at higher HA-1077 concentrations (Figures 4C,D and 5C,D).

### Induction of apoptosis by HA-1077

**Figure 6:**
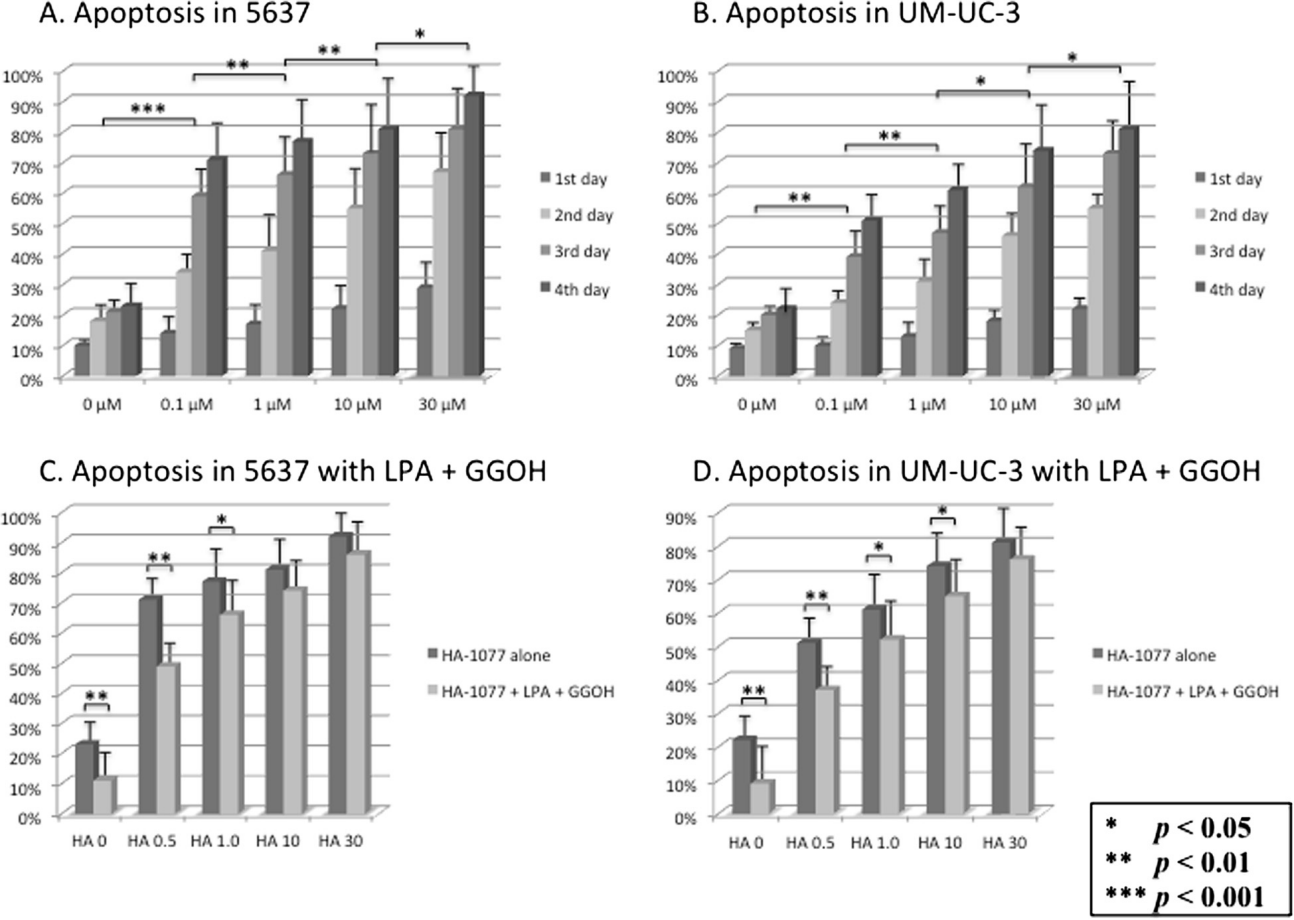
**The effects of HA-1077 (0.5–30** **μM) on bladder cancer cell viability.** Fluorescence images generated by terminal deoxynucleotidyltransferase-mediated UTP end-labeling (TUNEL) analysis of the bladder cancer cells. Total cell population was visualized by staining with 4^′^,6-diamidine-2-phenylin- dole (DAPI). Graph at right shows apoptotic index, the percentage of apoptotic cells in 1000 cells. HA-1077 demonstrates apoptosis induction of 5637 **(A)** and UM-UC-3 cells **(B)**. LPA plus GGOH reduced apoptotic cells in 5637 **(C)** and UM-UC-3 cells **(D)**. The data show the 95% confidential interval.

We examined the effect of HA-1077 on apoptosis of human bladder cancer cells in vitro. Addition of HA-1077 to cultured cells led to marked induction of apoptosis in a dose-dependent manner compared with control cultures, and this effect was seen for both HA-1077 alone and HA-1077 combined with LPA and GGOH (Figures 6A, B). When the difference in the percentage of apoptotic cells at each HA-1077 concentration was compared between cultures with HA-1077 alone and cultures with HA-1077 plus LPA and GGOH, it gradually decreased at higher concentrations of HA-1077 (Figures 6C, D).

### Influence of HA-1077 on LPA-induced cell migration

**Figure 7:**
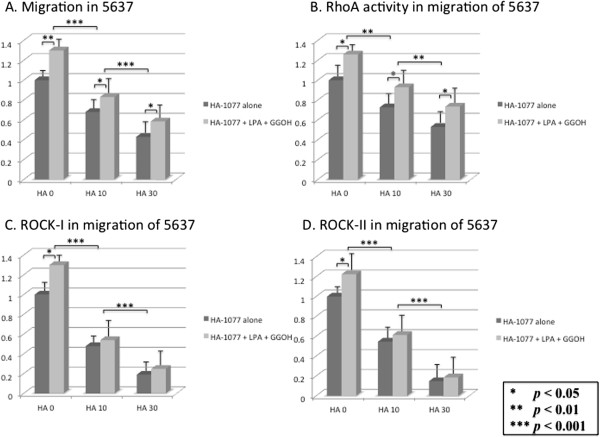
**The effects of HA-1077 (0–30** **μM) on migration in 5637 cell.** The cancer cells were incubated in supplemented with HA-1077 with/without LPA and GGOH. **(A)** The percentage of migrated cells decreased in dose-dependent manner. LPA and GGOH blocked HA-1077 induced suppression of migration. **(B-D)** RhoA activity was not statistically different between with and without HA-1077, but ROCK-I and ROCK-II expression was significantly reduced in the cells on the underside of each filter. The data show the 95% confidential interval.

**Figure 8:**
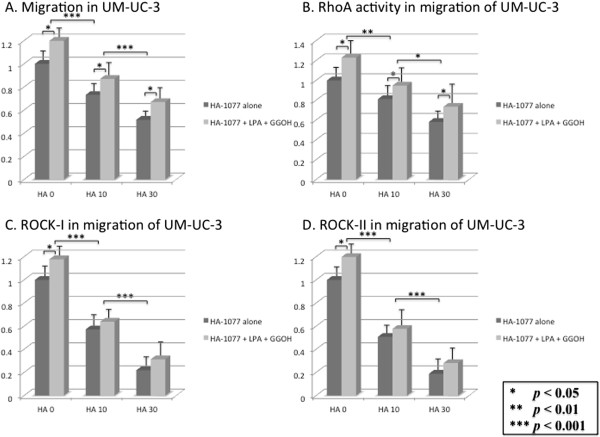
**The effects of HA-1077 (0–30** **μM) on migration in UM-UC-3 cell.** The cancer cells were incubated in supplemented with HA-1077 with/without LPA and GGOH. **(A)** The percentage of migrated cells decreased in dose-dependent manner. LPA and GGOH blocked HA-1077 induced suppression of migration.** (B-D)** RhoA activity was not statistically different between with and without HA-1077, but ROCK-I and ROCK-II expression was significantly reduced in the cells on the underside of each filter. The data show the 95% confidential interval.

We next examined the effect of HA-1077 on the migration of cultured human bladder cancer cells. Addition of LPA and GGOH increased the migration of bladder cancer cells compared with control cultures (Figures 7A and 8A). Cell migration was suppressed by HA-1077 in a dose-dependent manner, both in cultures with HA-1077 alone and in cultures with HA-1077 plus LPA and GGOH (Figures 7A and 8A). At the same time, RhoA activity and the expression of ROCK-I and ROCK-II were all significantly reduced by HA-1077 in a dose-dependent manner (Figures 7B-D and B-D). The dose-dependent down-regulation of the expression of these proteins by HA-1077 is likely to occur in parallel to the reduction in the number of migrating cells. Regarding changes of protein expression, the difference of ROCK-I and ROCK-II expression between cultures with HA-1077 alone and cultures with HA-1077 plus LPA and GGOH gradually decreased at higher concentrations of HA-1077 (Figures 7C, D and 8C, D). In contrast, RhoA activity was higher in the latter cultures at each HA-1077 concentration (Figures 7B, 8B).

## Discussion

Tumor cell migration is essential for metastasis, and metastasis is the most common fatal complication of cancer in humans. For the dissemination of tumor cells to distant organs to occur, migration of tumor cells through the fluid spaces of the body is essential [7]. Y-27632 [18] is another ROCK inhibitor that effectively suppresses tumor cell motility [19,20]. We previously reported that increased activity or overexpression of Rho and ROCK were associated with local invasion, metastasis, and an unfavorable prognosis of urogenital cancer including urothelial cancer [21-23], indicating that the Rho/ROCK pathway may be a potential target for anticancer therapy. However, there has been no reliable data regarding the effect on bladder cancer when ROCK is targeted by an inhibitor.

In the present study, LPA and GGOH induced an increase of cell proliferation that was correlated with increases of both RhoA activity and ROCK expression. The dose-dependent suppressive effect of HA-1077 on cell proliferation was accompanied by a marked decrease of ROCK-I and ROCK-II protein expression, while there was only a slight decrease of RhoA activity. This inhibitory effect of HA-1077 on cell proliferation was reduced by the addition of LPA and GGOH to cultures. On the other hand, RhoA activity was significantly higher in cultures with HA-1077 plus LPA and GGOH at each HA-1077 concentration, but the difference in the level of ROCK-I and ROCK-II protein expression between cultures with HA-1077 alone and cultures with HA-1077 plus LPA and GGOH gradually decreased at higher HA-1077 concentrations. These findings suggest that HA-1077 may selectively inhibit urothelial tumor cell proliferation via suppression of ROCK, but not by acting on RhoA.

The antiproliferative effect of HA-1077 was also evaluated using the clonogenic assay. The clonogenic cell survival assay is an effective method for the determination of single cell proliferation capacity, thereby retaining its reproductive ability to form a large colony or a clone [30,31]. We found that bladder cancer cells were less able to form colonies in response to exposure to HA-1077 compared to those without any treatment. These results suggested that HA-1077 inhibits the proliferation of bladder cancer cells at a certain rate.

With regard to apoptosis, HA-1077 caused the marked induction of apoptosis in a dose-dependent manner, but the difference in the percentage of apoptotic cells between cultures with HA-1077 alone and cultures with HA-1077 plus LPA and GGOH gradually became smaller at higher HA-1077 concentrations. This suggested that the pro-apoptotic effect of HA-1077 was more effective suppression of ROCK at higher concentrations of HA-1077.

In the cell migration study, addition of LPA and GGOH increased the migration of human bladder cancer cells. Cell migration was suppressed by HA-1077 in a dose-dependent manner, while this suppressive effect of HA-1077 was inhibited by addition of LPA and GGOH. Western blotting analysis of the cells from the underside of each filter showed that RhoA activity and ROCK-I and ROCK-II expression were significantly decreased by HA-1077 in a dose-dependent manner. This dose-dependent inhibition of these proteins by HA-1077 is likely to occur in parallel with a reduction in the number of migrating cells, since RhoA activity was higher in cultures with HA-1077 plus LPA and GGOH at each HA-1077 concentration, while expression of ROCK-I and ROCK-II did not increase.

LPA increases GTP loading, while GGOH activates geranylgeranylation. The mevalonate pathway is required for geranylgeranylation of Rho by GGOH. After Rho has been activated by geranylgeranylation, its downstream effector ROCK is activated when it selectively binds to the active GTP-bound form of Rho. In the present study, addition of LPA and GGOH to cultured cells increased RhoA activity and up-regulated the expression of ROCK-I and ROCK-II, while HA-1077 dramatically suppressed both ROCK-I and ROCK-II dramatically, but did not reduce RhoA activity. These findings indicate that HA-1077 may selectively inhibit bladder cancer cell proliferation and migration via suppression of ROCK, but not by blocking RhoA activity.

It is important to study signaling cross-talk between ROCK and other downstream effectors in the Rho family of GTPases. ROCK belongs to the AGC (protein kinase A/protein kinase G/protein kinase C) family of serine-threonine kinases. ROCK-I and ROCK-II share 65% overall identity, with 87% identity of the kinase domain [11,36,37]. We previously reported that overexpression of both ROCK-I and ROCK-II was associated with poor differentiation, invasiveness, metastasis, and an unfavorable prognosis of human bladder cancer [21]. In the present study, we found that the expression of both ROCK-I and ROCK-II protein was decreased by HA-1077. However, since HA-1077 and Y-27632 are not highly selective for ROCK-I and ROCK-II, when ROCK-I and ROCK-II were suppressed by HA-1077, downstream molecules such as the myosin binding subunit of the myosin light chain (MLC) phosphatase (MYPT)-1 and LIN-11, Isl1, and MEC-3 domain kinase (LIMK) might compensate for ROCK inhibition [38].

Numerous downstream effectors are involved in the Rho signaling pathway [39]. p140mDia is a mammalian homologue of *Drosophila* diaphanous that controls actin polymerization [40]. ROCK and p140mDia act cooperatively during stress fiber formation to mediate the effects of Rho [41]. Arakawa et al. [42] showed that the direction of Rho signaling is dependent on the local level of Rho-GTP, since a high Rho-GTP level induces ROCK activation and a low level preferentially activates mDia and induces Rac activation. Although we did not investigate Rac, Cdc42, and mDia in the present study, Rac1 activity was increased in cancers of the human upper urinary tract and was related to tumor progression in our previous study [23]. Furthermore, HA-1077 did not influence RhoA activity in the current study. Rho family GTPases, including Rho, Rac, and Cdc42, have been shown to differentially and cooperatively contribute to triggering invasive behavior by tumor cells [43]. Taken together, these findings suggest that if ROCK is suppressed by a ROCK inhibitor, other signaling pathways (including LIMK, Rho, Rac, Cdc42, and mDia) might be activated to compensate for ROCK inhibition.

However, HA-1077 may still be an attractive candidate. As described above, ROCK belongs to the AGC protein kinase family of serine-threonine kinases [11,36,37], so HA-1077 might have a nonspecific inhibitory effect on other protein kinases from this family [44]. Mutation and/or dysregulation of these AGC protein kinases contributes to the pathogenesis of human cancer [45,46]. Recently, Nakabayashi et al. reported that HA-1077 suppresses neovascularization and tumor growth, in association with reduced expression of VEGF, matrix metalloproteinase (MMP)-2, and MMP-9, as well as attenuating the phosphorylation of extracellular signal-regulated kinase 1 and 2 (ERK1/2) and DNA binding activity of activator proteins (a key downstream transcriptional factor for ERK1/2) in malignant glioma cells, indicating that the anti-angiogenic effect of HA-1077 may be due to the combined inhibition of ROCK and the mitogen-activated protein kinase kinase (MEK)/ERK pathway [47].

The Rho/ROCK pathway is known to play an important role in the progression of cancer. The present findings indicate that HA-1077 prevents the proliferation and migration of bladder cancer cells and also induces apoptosis by inhibiting ROCK, suggesting that ROCK may be an attractive molecular target agent for anticancer therapy. However, this study did not show that HA-1077 was equally effective in animal models of bladder cancer developed with the 5637 or UM-UC-3 bladder cancer cell lines. Rath et al. suggested that a translational approach to ROCK signaling is necessary for clinical development of ROCK inhibitors to treat cancer and they identified the following issues to be addressed: 1) the tissue and tumor patterns of ROCK expression/activity, 2) the mode of ROCK inhibition, 3) the inhibition of ROCK targets and parallel pathways, 4) the influence of combination therapy, and 5) development of drugs targeting the extracellular matrix of tumors [38]. In order to directly address these issues, we should compare the effectiveness of HA-1077 and its vehicle control in vivo by developing a mouse model of human bladder cancer in the future.

Improved understanding of how Rho family GTPases and their downstream effectors mutually and specifically interact in human cancers may throw more light on the best therapeutic approach to cancer and might lead to new treatment protocols.

## Conclusions

The Rho family of small GTP-binding proteins and its best-characterized downstream effector, ROCK, are known to have an important influence on the regulation of cell motility and play a pivotal role in tumor progression. The ROCK inhibitor HA-1077 effectively inhibits tumor cell proliferation and migration and also induces apoptosis. The present study investigated the effects of this ROCK inhibitor on the growth, migration, and apoptosis of bladder cancer cells. To explore the underlying molecular mechanisms, we treated bladder cancer cells with HA-1077 and then examined changes of the GTP-bound active form of RhoA and expression of its downstream effector ROCK. Treatment with HA-1077 caused a decrease in the growth and migration of bladder cancer cells, while apoptosis showed a significant increase. Expression of ROCK-I and -II proteins was decreased by exposure of tumor cells to HA-1077, while RhoA activity was not affected. These findings indicate that HA-1077 prevents the proliferation and migration of bladder cancer cells and also induces apoptosis by inhibiting ROCK, suggesting that ROCK may be a molecular target for the treatment of cancer.

## Competing interests

The authors declare that they have no competing interests.

## Authors’ contributions

HA, TK* and NA initiated the study, participated in its design and coordination, carried out the study, performed the statistical analysis. HA and TK * drafted the manuscript. KH, TM, YY, AM, HY, HB, MY and YF carried out the study. HS and K-IY participated in the design of the study and helped to draft the manuscript. All authors read and approved the final manuscript.

## Pre-publication history

The pre-publication history for this paper can be accessed here:

http://www.biomedcentral.com/1471-2407/14/412/prepub
